# Increased neutrophil counts are associated with poor overall survival in patients with colorectal cancer: a five-year retrospective analysis

**DOI:** 10.3389/fimmu.2024.1415804

**Published:** 2024-09-23

**Authors:** Libia Alejandra Garcia-Flores, María Teresa Dawid De Vera, Jesus Pilo, Alejandro Rego, Gema Gomez-Casado, Isabel Arranz-Salas, Isabel Hierro Martín, Julia Alcaide, Esperanza Torres, Almudena Ortega-Gomez, Hatim Boughanem, Manuel Macias-Gonzalez

**Affiliations:** ^1^ Department of Endocrinology and Nutrition, Virgen de la Victoria University Hospital, Málaga, Spain; ^2^ Institute of Biomedical Research in Malaga (IBIMA)-Bionand Platform, University of Malaga, Málaga, Spain; ^3^ Unidad de Gestión Clínica Intercentros (UGCI) de Anatomía Patológica, Instituto de Investigación Biomédica de Málaga (IBIMA), Hospital Universitario Virgen de la Victoria, Universidad de Málaga, Málaga, Spain; ^4^ Medical Oncology Service, Hospital Regional Universitario de Málaga, Biomedical Research Institute of Malaga (IBIMA), Málaga, Spain; ^5^ Unidad de Gestión Clínica Intercentros (UGCI) de Oncología Médica, Instituto de Investigación Biomédica de Málaga (IBIMA), Hospital Universitario Virgen de la Victoria, Málaga, Spain; ^6^ Centro de Investigación Biomédica en Red (CIBER) Fisiopatologia Obesidad y Nutricion (CIBEROBN), Instituto de Salud Carlos III, Madrid, Spain; ^7^ Lipids and Atherosclerosis Unit, Department of Internal Medicine, Hospital Universitario Reina Sofia, Cordoba, Spain; ^8^ Maimonides Institute for Biomedical Research in Cordoba (IMIBIC), Cordoba, Spain

**Keywords:** colorectal cancer, neutrophils, overall survival, prognosis, inflammation

## Abstract

**Background:**

Colorectal cancer (CRC) continues to be a major health concern in today’s world. Despite conflictive findings, evidence supports systemic inflammation’s impact on CRC patients’ survival rates. Therefore, this study aims to assess the prognostic role of the innate immune system in patients with CRC.

**Method:**

A total of 449 patients were included, with a 5-year follow-up period, and absolute neutrophil counts and their related ratios were measured.

**Results:**

The non-survival group had increased levels of white blood cells, neutrophils (both *p*<0.001), and monocytes (p=0.038), compared to the survival group, along with other neutrophil-related ratios. We observed increased mortality risk in patients in the highest tertile of white blood cells [HR=1.85 (1.09-3.13), p<0.05], neutrophils [HR=1.78 (95% CI: 1.07-2.96), p<0.05], and monocytes [HR=2.11 (95% CI: 1.22-3.63)], compared to the lowest tertile, after adjusting for all clinicopathological variables. Random forest analysis identified neutrophils as the most crucial variable in predicting survival rates, having an AUC of 0.712, considering all clinicopathological variables. A positive relationship between neutrophil counts and metastasis was observed when neutrophil counts are considered continuous (β=0.92 (0.41), p<0.05) and tumor size (width) when neutrophils were considered as logistic variable (T1 vs T3) [OR=1.42, (95% CI: 1.05-1.98), p<0.05].

**Conclusion:**

This study offers comprehensive insights into the immune factors that impact the prognosis of CRC, emphasizing the need for personalized prognostic tools.

## Introduction

Colorectal cancer (CRC), accounts for 10% of all cancer diagnoses and is the third most commonly diagnosed, as well as the second leading cause of cancer-related deaths worldwide (1). While enhanced screening has improved survival rates, the five-year survival rate for advanced CRC remains approximately 20%, largely dependent on the tumor stage (2). The health impact of CRC is largely attributed to systemic inflammation, a key factor in its development and progression (3, 4). Inflammatory tumors in CRC are linked to lower survival rates after relapse, highlighting the significance of understanding the role of inflammation role in CRC (5). Recent research has turned to circulating inflammatory markers, particularly neutrophils, as indicators for cancer prognosis and progression (6, 7). Specifically, neutrophils have been strongly associated with various cancer outcomes (8). For instance, a systematic review and meta-analysis encompassing 71 publications and 32,788 patients confirmed that an NLR (neutrophil-to-lymphocyte ratio) was associated with poor patient outcomes, including overall survival (Hazard Ratio (HR) = 1.84, 95% CI: 1.68 – 2.03) and surrogate endpoints, such as disease recurrence and progression-free survival (HR = 1.72, 95% CI: 1.51 – 1.95) (9). Other immune biomarkers, such as interleukin 8 (IL8) and LMR (lymphocyte-to-monocyte ratio) have also been identified as significant prognostic factors (10), suggesting the utility of immune system biomarkers in predicting the prognosis of CRC. Despite these findings, the complete potential of these markers in CRC prognosis is not fully comprehended, and the dual role of neutrophils in CRC complicates their prognostic value (11). Therefore, it is crucial to understand their potential role in predicting CRC survival, to establish preventive and follow-up strategies.

In this study, we hypothesized that inflammatory and immune system biomarkers could be potential indicators for predicting the outcome in CRC patients. This retrospective study aims to examine the relevance of specific routine inflammatory and immune system biomarkers in predicting the survival of patients with CRC and to explore clinicopathological variables that could impact these biomarkers. In addition, our objective was also to develop comprehensive predictive models for overall survival in CRC patients, which could improve patient management and healthcare outcomes by enhancing predictive accuracy.

## Materials and methods

### Study design and patients included in the study

**Figure 1:**
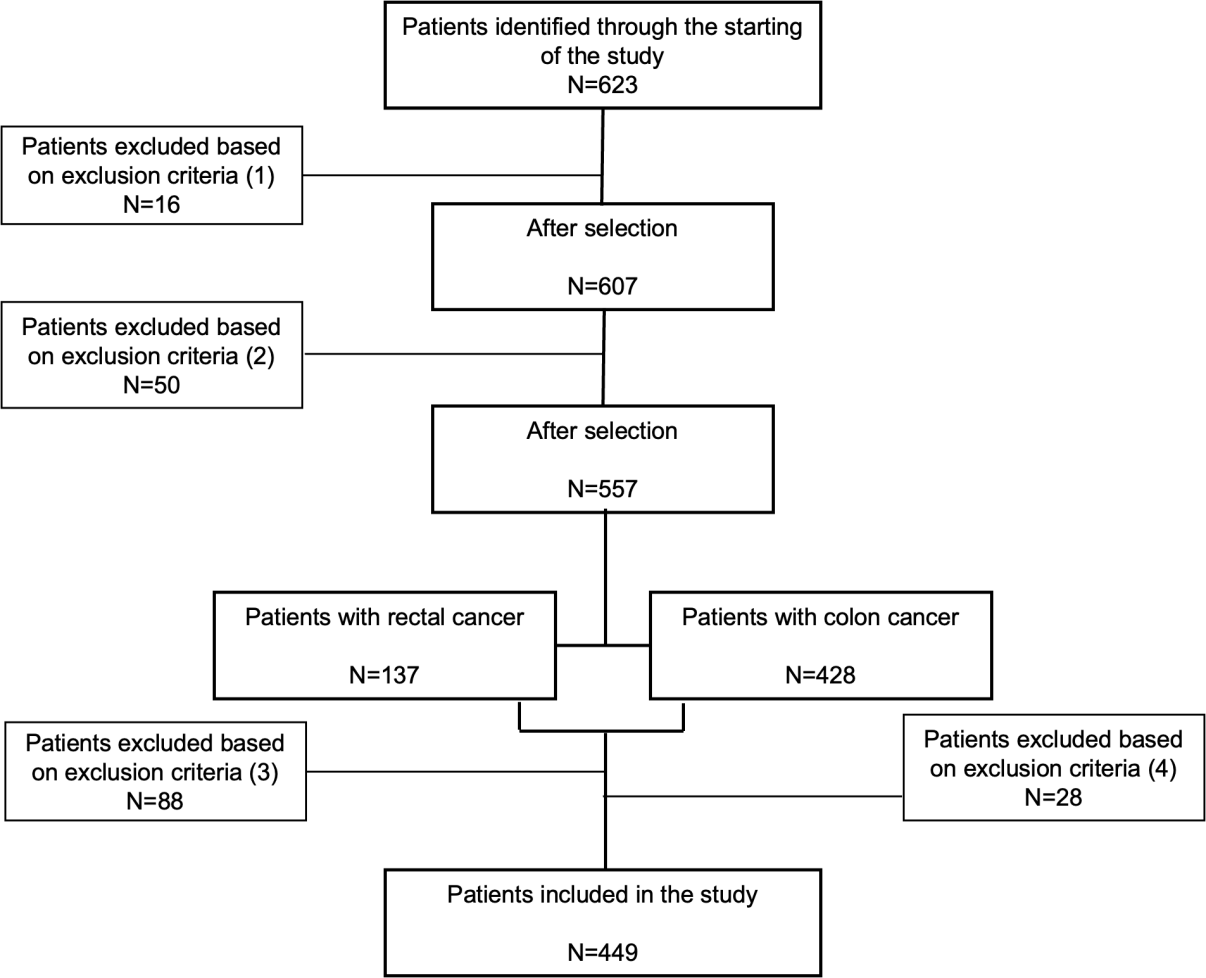
Flow diagram of patient selection for inclusion in our study. A total of 623 participants were initially identified as potential candidates. Exclusions were made for the following reasons: (1) sixteen participants from 1997 to 2011 were excluded due to incomplete histopathologic diagnostic records; (2) fifty patients were excluded due to missing clinicopathological data; (3) eighty-eight rectal cancer patients and (4) twenty-eight colon cancer patients were excluded because they had received neoadjuvant treatment (radiotherapy, chemotherapy, or both) before surgery, or their vital status was unconfirmed. Ultimately, 449 patients were included in the final analysis.

This study included 623 patients diagnosed with CRC at Virgen de la Victoria University Hospital, Málaga, Spain, between January 1999 to May 2017. Patients included in the study were diagnosed with CRC through colonoscopy and biopsy, with comprehensive medical records and pathological examinations available. Biopsy samples were classified histologically according to the World Health Organization’s criteria (12). After selecting patients diagnosed with CRC, the majority underwent tumor resection surgery. Surgical procedures included hemicolectomy, lower anterior resection, and total meso-colorectal excision, often involving ileostomy. Specifically, among patients with stage I and II cancer, 97.5% underwent tumor resection, with only 2.5% not undergoing surgery. For stage III, 96.0% had their tumors removed, while 4.0% did not. However, patients in stage IV included various procedures such as hemicolectomy, metastasectomy, and sigmoidectomy. All patients selected for inclusion had not received any adjuvant treatment prior to surgery (**Figure 1**). Post-surgery, patients were monitored for a minimum of five years, with follow-up visits every three months in the first two years and every six months thereafter, including physical exams, biochemical assays, and colonoscopies. The study complied with the Declaration of Helsinki and was approved by the ethics committee (PEIBA: 0582-N-23) of University Hospital “Virgen de la Victoria”, Málaga, Spain, following relevant guidelines and regulations.

### Samples included in the study

The study involved collecting blood samples from all participants. After an overnight fast, venous blood samples were obtained from the median cubital vein. These samples were collected within 24 hours before surgery to ensure they reflected the preoperative immune status of the patients. In some cases, blood samples were taken up to a month before surgery due to medical reasons for delaying the operation. Serum samples were obtained through centrifugation of blood samples at 4,000 r.p.m. for 15 minutes at 4°C.

Serum levels of fasting glucose, total cholesterol, triglycerides, high-density lipoprotein (HDL), and Low-density lipoprotein (LDL) cholesterol (calculated using the Friedewald equation) were assessed with a Dimension Autoanalyzer (Dade Behring Inc., Deerfield, IL, USA). Fasting insulin levels were measured through radioimmunoassay (BioSource International Inc. (Camarillo, CA, USA)), and insulin resistance was calculated using the homeostasis model assessment (HOMA-IR) formula: HOMA-IR = fasting insulin (IU/mL) × fasting glucose (mmol/L)/22.5. Flow cytometry analyzed various blood components including basophils, eosinophils, lymphocytes, monocytes, neutrophils, and platelets, and several ratios (**Supplementary Table 1**) were computed to evaluate systemic inflammation.

### Statistical analysis

This study utilized descriptive statistics to analyze demographic and clinical data, presenting continuous variables with mean and standard deviation (SD), and categorical data as frequencies and percentages. The Mann-Whitney and Chi-squared tests compared categorical data. Survival data were analyzed using Kaplan-Meier curves and Cox regression. Linear and logistic regressions examined the relationship between neutrophil counts and cancer outcomes, calculating odds ratios. To identify the most significant predictors of mortality, a random forest analysis was performed using mean decrease in accuracy (MDA). Finally, the prognostic performance of the model was evaluated by determining the Area Under the Curve (AUC) in the receiver operating characteristic (ROC) analysis. Statistical analyses were conducted using SPSS 26.0 (Chicago, IL, USA) and R v3.5.1(R Foundation for Statistical Computing, Vienna, Austria), with a significance threshold of p<0.05.

## Results

### Baseline clinicopathological variables of the patients included in the study

The study included 449 patients with CRC (**Figure 1**). Patients were categorized based on their survival status following a 5-year follow-up period. Among them, 262 patients with CRC were recorded as survivors, while 196 patients did not survive. **Table 1** summarizes the characteristics and clinicopathological features of the patients included in the study. We found that the non-survival group was significantly older than the survival group (*p*<0.001). When comparing tumor stages (TNM classification) between survival and non-survival groups significant differences were observed. A majority of non-survival group patients were in advanced stages of the disease (28.1% in stage III and 46.4% in stage IV). In contrast, only 5% of the survival group patients were in stage IV, with a higher proportion in the earlier stages (I and II) (*p*<0.001). Moreover, a higher percentage of patients in the non-survival group (34.1%) had high-grade histology compared to the survival group (19.9%) (*p*=0.005). In addition, metastasis was present in 51.2% of the non-survival group, compared to just 9.46% in the survival group (*p*<0.001). Disease recurrence also showed a marked disparity, occurring in 30.5% of the non-survival group versus 9.19% of the survival group (*p*<0.001).

**Table 1:** Baseline characteristic of patients with colorectal cancer divided by survival and non-survival patients, including biochemical and clinicopathological variables.

Variables	Survival	Non-survival	*p* value
	*N=262*	*N=196*	
Age, years	67.1 (11.9)	71.8 (11.4)	<0.001***
Sex, (Males vs Females):			0.098
Males,	138 (54.5%)	123 (62.8%)	
Females,	115 (45.5%)	73 (37.2%)	
Weight, kg	73.9 (12.9)	72.2 (14.5)	0.256
Body-mass index, kg/m²	27.6 (4.39)	27.5 (6.47)	0.855
Fasting glucose plasma, mg/dL	110 (38.5)	119 (58.8)	0.055
HbA1c, %	15.7 (27.9)	6.61 (1.45)	0.356
Total cholesterol, mg/dL	178 (45.9)	169 (47.9)	0.309
Triglycerides, mg/dL	133 (76.1)	134 (58.7)	0.955
HDL cholesterol, mg/dL	46.4 (12.9)	41.1 (16.0)	0.304
LDL cholesterol, mg/dL	107 (45.8)	105 (45.7)	0.867
Type 2 Diabetes Mellitus, %:			1.000
No	187 (73.9%)	144 (73.5%)	
Yes	66 (26.1%)	52 (26.5%)	
Alcohol consumption, %:			0.300
No	142 (68.9%)	114 (74.5%)	
Yes	64 (31.1%)	39 (25.5%)	
Smoking, %:			0.499
No	198 (85.0%)	136 (81.9%)	
Yes	35 (15.0%)	30 (18.1%)	
Familial history of colorectal cancer			0.096
No	126 (49.8%)	114 (58.2%)	
Yes	127 (50.2%)	82 (41.8%)	
Tumor site:			0.235
Rectum	32 (12.6%)	17 (8.67%)	
Colon	221 (87.4%)	179 (91.3%)	
Tumor grade histology:			0.060
G1	93 (42.5%)	44 (30.8%)	
G2	101 (46.1%)	71 (49.7%)	
G3	21 (9.59%)	23 (16.1%)	
Tumor stage, TNM			<0.001***
I	8 (4.42%)	4 (2.61%)	
II	83 (45.9%)	35 (22.9%)	
III	81 (44.8%)	43 (28.1%)	
IV	9 (4.97%)	71 (46.4%)	
Histology grade			0.005**
Low grade	164 (80.0%)	89 (65.9%)	
High grade	41 (20.0%)	46 (34.1%)	
Tumor size width, cm	4.54 (1.78)	4.47 (2.01)	0.807
Tumor size large, cm	3.53 (1.66)	3.30 (1.66)	0.387
Metastasis:			<0.001***
No	201 (90.5%)	81 (48.8%)	
Yes	21 (9.46%)	85 (51.2%)	
Chemotherapy:			0.698
No	139 (55.2%)	102 (52.8%)	
Yes	113 (44.8%)	91 (47.2%)	
Radiotherapy:			0.277
No	217 (94.3%)	140 (90.9%)	
Yes	13 (5.65%)	14 (9.09%)	
Disease recurrence:			<0.001***
No	165 (90.7%)	73 (69.5%)	
Yes	17 (9.34%)	32 (30.5%)	
*KRAS* mutation:			0.062
No	22 (64.7%)	32 (43.2%)	
Yes	12 (35.3%)	42 (56.8%)	

Data are represented as mean (SD) or n (%). Groups were divided according to survival outcomes after 5-years of follow-up. Asterisk indicates significant difference between groups according to the Mann Whitney test and Chi squared test was used for variables expressed as percentage (***p<0.001, **p<0.01). Histologic Type groups: G1.-Polypoid, pedunculated, Exophytic, Ulcerated or Ulcerated-central, Coliform and Vegetative, G2.-Necrotic, Ulcerated, and Ulcerated-necrotic, G3.-Mucinous, and G4.-Stenotic and Stenotic-ulcerated. Histologic Type Groups and TNM stage categories were based on the protocol for examination of resection specimens from patients with primary Carcinoma of the colon and rectum by the College of American Pathologists version 4.2.0.0, 2021. Tumor site includes colon: right-sided and left-sided colon, as well as transversal colon. HbA1c, Hemoglobin glycosylated; HDL, high density lipoprotein; KRAS, Kirsten Rat Sarcoma Viral Oncogene Homolog; LDL, low density lipoprotein.

### Baseline circulating leukocytes of the patients included in the study

To assess the impact of systemic inflammation on overall survival, we analyzed leukocyte counts and inflammatory variables in our study cohort. We observed that the non-survival group had higher levels of white blood cell (p<0.001), neutrophil (p<0.001), monocyte (p=0.035), and platelet (p=0.003) counts compared to the survival group (**Table 2**). Additionally, we observed lower levels of circulating albumin in the non-survival group compared to the survival group (p=0.007). Moreover, various ratios derived from circulating immune cells demonstrated higher values in the non-survival group than in the survival group, including NER (p<0.017), NLR (p<0.001), NPR (p=0.039), NWR (p=0.006), PLR (p=0.001), and LMR (p=0.001) (**Table 2**).

**Table 2:** Baseline inflammatory and immune profile of patients with colorectal cancer divided by survival and non-survival patients.

Variables	Survival	Non-survival	*p* value
	*N=252*	*N=193*	
White blood cells, 10³/µL	7.29 (2.44)	8.69 (3.59)	<0.001***
Neutrophils, 10³/µL	4.85 (2.17)	6.13 (3.38)	<0.001***
Monocytes, 10³/µL	0.55 (0.50)	0.63 (0.32)	0.038*
Lymphocytes, 10³/µL	1.68 (0.54)	1.64 (0.63)	0.502
Platelets, 10³/µL	282 (108)	314 (118)	0.003**
NER,	32.4 (21.7)	38.8 (28.7)	0.011*
NLR,	3.22 (2.02)	4.48 (3.69)	<0.001***
NMR,	10.7 (9.88)	10.5 (5.18)	0.726
NPR,	0.02 (0.01)	0.02 (0.01)	0.021*
NWR,	0.65 (0.09)	0.68 (0.11)	0.004**
NBR,	99.9 (67.0)	134 (156)	0.006**
PLR,	184 (92.0)	221 (123)	<0.001***
LMR,	3.89 (3.23)	3.11 (1.79)	0.001**
hsCRP, mg/dL	53.9 (68.1)	73.1 (91.0)	0.213
Albumin, mg/dL	3.43 (0.56)	3.12 (0.56)	0.007**

Data are represented as mean (SD) or n (%). Groups were divided according to survival outcomes after 5-years of follow-up. Asterisk indicates significant difference between groups according to the Mann Whitney test and Chi squared test was used for variables expressed as percentage (***p<0.001, **p<0.01, *p<0.05). hsCRP, High-sensitive C reactive protein; NBR, Neutrophil-to-basophil Ratio; NER, Neutrophil-to-eosinophil Ratio; NERR, Neutrophil-to-erythrocyte Ratio; NHR, Neutrophil-to-HDL Ratio; NMR, Neutrophil-to-monocyte ratio; NLR, Neutrophil-to-lymphocyte ratio; NPR, Neutrophil-to- platelet ratio; LDL, light density lipoprotein.

### Association between inflammatory variables and the risk of mortality

**Figure 2:**
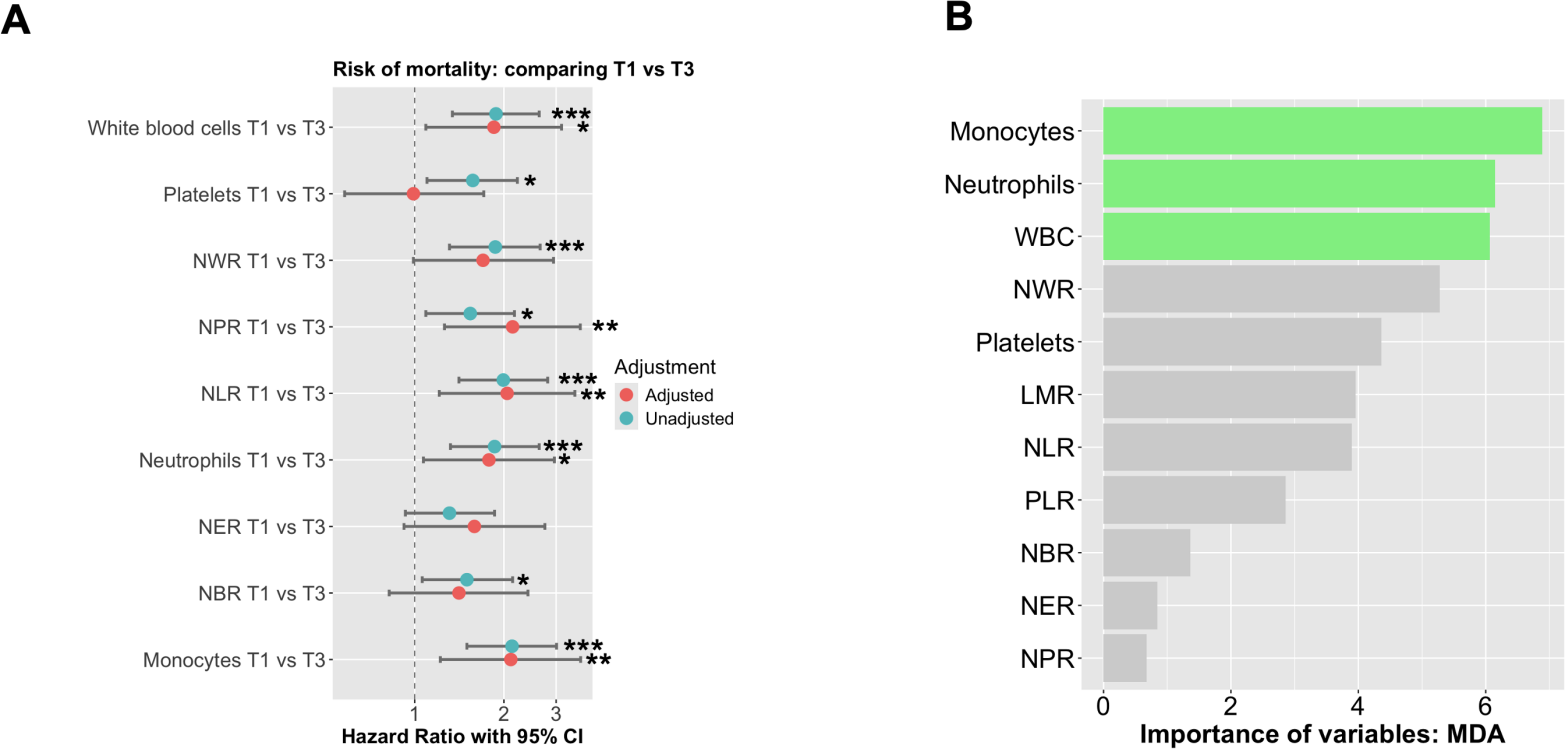
The probability of mortality according to absolute neutrophil counts was analyzed using Cox proportional hazards regression. **(A)** The analysis was performed using Cox regression curves with tertiles of absolute neutrophil counts at baseline, both unadjusted and adjusted for BMI, sex, age, chemo- and radiotherapy, cancer stage (I+II vs III+IV), tumor site (colon vs rectum) + Histological grade (low vs high grade). T1 represents low neutrophil levels, T2 represents intermediate levels, and T3 represents high neutrophil levels. **(B)** Random forest of all significant variables included in our study to predict mortality. The symbols *, **, and *** indicate statistical significance, where * represents p < 0.05, ** represents p < 0.01, and *** represents p < 0.001.

To evaluate the correlation between inflammatory variables and the risk of mortality, a logistic regression analysis was performed. Patients were stratified into tertiles based on baseline absolute neutrophil counts and other ratios. Tertile 1 (T1) comprised patients with the lowest neutrophil counts, Tertile 2 (T2) included those with intermediate counts, and Tertile 3 (T3) consisted of patients with the highest counts. Following adjustments for BMI, sex, age, chemotherapy, radiotherapy, cancer stage (I+II vs. III+IV), tumor site (colon vs. rectum), and histological grade (low vs. high grade), we observed that patients in T3 of white blood cells [HR=1.85 (1.09-3.13), p<0.05], NPR [HR=2.14 (95% CI: 1.21-3.47), p<0.01], NLR [HR=2.05 (95% CI: 1.21-3.47), p<0.01], neutrophils [HR=1.78 (95% CI: 1.07-2.96), p<0.05], and monocytes [HR=2.11 (95% CI: 1.22-3.63), p<0.01] had an increased risk of mortality when compared to those in T1 (**Figure 2A**). To determine the significant variables for predicting mortality within our population, we performed a random forest analysis. Monocytes, neutrophils, and white blood cells emerged as the most crucial factors in predicting mortality (**Figure 2B**).

### Immune system variables and overall survival rate

**Figure 3:**
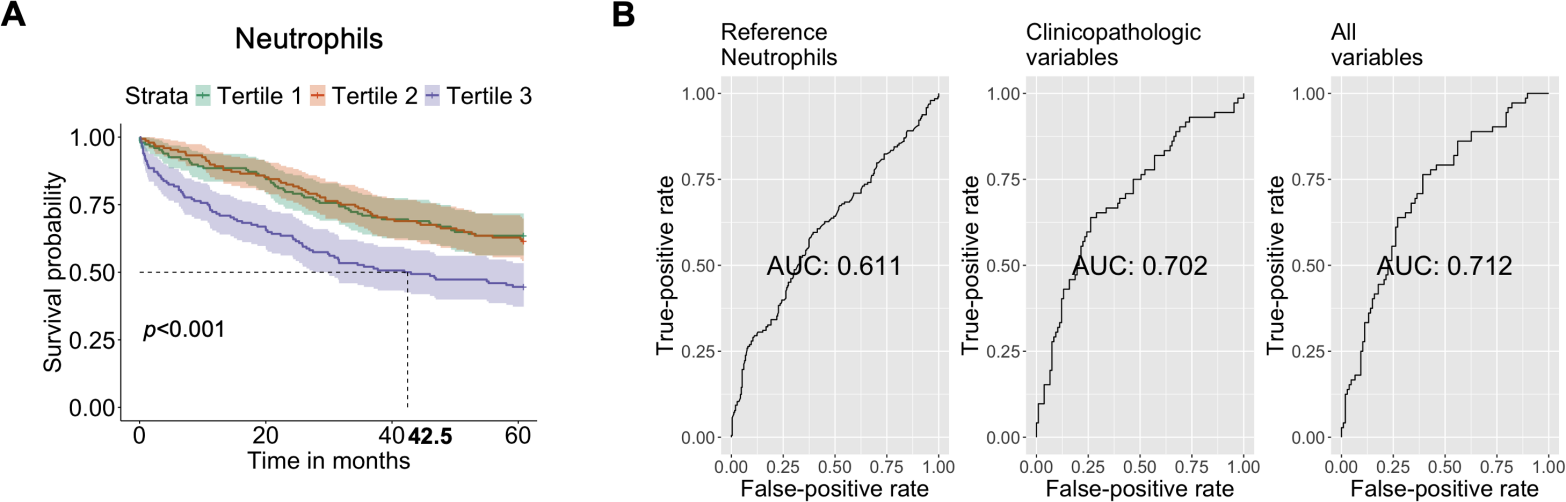
Prognostic Value of Hematological Parameters and Clinical-pathologic Variables in Predicting Mortality in CRC. **(A)** Cox regression analysis by comparing T1 and T2 vs T3, to predict the risk of mortality using neutrophil-related ratios. **(B)** Three Receiver Operating Characteristic (ROC) curves with corresponding Areas Under the Curve (AUC).

To assess the value of these variables as predictive tools for overall survival in our population, Kaplan-Meier curves derived from Cox regression were generated. We observed that patients within T3 of neutrophils had poorer survival, with a median of 42.5 months, compared to both T2 and T1 (both with p<0.001) (**Figure 3A**). Additionally, to evaluate the prognostic value of neutrophils, ROC curves were constructed. When analyzing neutrophils alone, the AUC was 0.611. Incorporating clinicopathological variables increased the AUC to 0.702. However, considering all variables together further improved the AUC to 0.712 (**Figure 3B**). Similar patterns were observed for monocytes and white blood cells. Patients within T3 of monocytes and white blood cells had poorer survival, compared to both T2 and T1 (both with p<0.001) (**Supplementary Figures 1A, C**). Additionally, to evaluate the prognostic value of monocytes and white blood cells, the AUC was 0.616 and 0.611, respectively. Incorporating clinicopathological variables increased the AUC to 0.714 and 0.710, respectively (**Supplementary Figures 1B, D**).

Finally, we conducted an analysis to identify factors influencing neutrophil counts, utilizing both linear and logistic regressions. After adjusting by age, sex, BMI, familial history of cancer, smoking, alcohol consumption and the presence of type 2 diabetes mellitus, the results showed that there is a positive relationship between neutrophil counts and the occurrence of metastasis when neutrophil counts are considered continuous (β=0.92 (0.41), p<0.05) and tumor size (width) when neutrophils were considered as logistic variable (T1 vs T3) [OR=1.42, (95% CI: 1.05-1.98), p<0.05] (**Table 3**).

**Table 3:** Logistic and linear regression analysis to predict those clinicopathological variables that are associated with neutrophil counts.

Variables	Variables	Neutrophils (continuous)	Neutrophils (T1 vs T3)
		β (SD)	OR (95% CI)
Tumor site (colon vs rectum)	Unadjusted	0.39 (0.45)	1.28 (0.58 – 2.88)
	Adjusted	0.40 (0.60)	1.89 (0.06 – 22.11)
Tumor stage (I+II vs III+IV)	Unadjusted	0.53 (0.33)	1.18 (0.69 – 2.01)
	Adjusted	0.59 (0.41)	1.39 (0.89 – 2.84)
Tumor size, width (cm)	Unadjusted	0.27 (0.11)*	1.44 (1.18 – 1.83)***
	Adjusted	0.13 (0.14)	1.42 (1.05 – 1.98)*
Tumor size, large (cm)	Unadjusted	0.33 (0.13)*	1.51 (1.18 – 2.02)**
	Adjusted	0.11 (0.17)	1.33 (0.99 – 1.88)
Metastasis (No vs Yes)	Unadjusted	1.19 (0.32)***	1.92 (1.12 – 3.31)*
	Adjusted	0.92 (0.41)*	1.97 (0.96 – 4.08)
Chemotherapy (No vs Yes)	Unadjusted	0.45 (0.27)	0.99 (0.63 – 1.58)
	Adjusted	0.74 (0.37)	1.13 (0.57 – 2.25)
Radiotherapy (No vs Yes)	Unadjusted	0.59 (0.58)	1.33 (0.53 – 3.41)
	Adjusted	0.43 (0.74)	0.78 (0.21 – 2.69)
Chemotherapy + Radiotherapy (No vs Yes)	Unadjusted	0.53 (0.26)*	1.04 (0.66 – 1.64)
	Adjusted	0.72 (0.37)	1.10 (0.56 – 2.19)
Histology grade (Low vs high grade)	Unadjusted	-0.21 (0.36)	1.01 (0.55 – 1.83)
	Adjusted	-0.41 (0.44)	0.78 (0.36 – 1.66)
Recurrence (Yes vs no)	Unadjusted	-0.21 (0.36)	0.63 (0.26 – 1.47)
	Adjusted	-0.39 (0.44)	0.55 (0.14 – 1.76)

Linear regression analysis using neutrophil counts as continuous variable and by comparing the highest vs the lowest tertiles of neutrophils as categoric variables. Adjusted model was adjusted for age, gender, BMI, familial history, smoking history, alcohol consumption and the presence of type 2 diabetes mellitus. Asterisk indicates significant difference between groups (***p<0.001, **p<0.01, *p<0.05).

## Discussion

In this retrospective study, we examined the survival outcomes of patients with CRC over five years. The study focused on assessing inflammatory and immune system biomarkers as prognostic indicators among both survivors and non-survivors. Our findings suggest that several biomarkers associated with neutrophils and their related ratios are effective predictors of overall survival in CRC patients. Particularly, neutrophils and monocytes emerged as the most significant predictors in the random forest model. Utilizing tertiles based on neutrophil counts, we observed that highest tertile of neutrophils at baseline showed notably poorer overall survival rates compared to the middle and the lowest tertiles. This observation yielded an AUC of 0.712, after incorporating all relevant clinicopathological variables, suggesting the potential of neutrophils and other variables as valuable prognostic indicators for predicting overall survival in patients with CRC. This study also highlights the potential role of neutrophils in cancer progression and disease relapse, warranting further research to clarify these observations.

Several biomarkers have been identified as potential indicators for diagnosis, prognosis, and treatment response in CRC (13). Specifically, there is an emerging predictive biomarker for cancer prognosis, which includes immune-related biomarkers for managing patients with CRC (13, 14). Laboratory markers of systemic inflammatory response have been extensively studied as prognostic and predictive tools in CRC (15). Neutrophils and related markers, such as NLR, have been widely proposed as diagnostic and prognostic biomarkers in several studies (16–18), as well as for overall survival in CRC (16, 19, 20). In our study, Cox logistic regression analysis revealed that increased NLR values were associated with poorer survival rates in CRC patients, even after adjusting for all relevant clinicopathological variables. This indicates the potential of NLR as a biomarker in CRC. Furthermore, other immune cell parameters, such as neutrophil and monocyte counts, as well as other ratios, showed reliable predictive utility, suggesting the involvement of the immune system in cancer progression and survival outcomes. However, in our study, a random forest analysis proposed neutrophils and monocytes as the most important predictor of overall survival, which supports previous findings, highlighting a mechanistic role in CRC.

When comparing neutrophil tertiles, the highest tertile displayed lower overall survival rates compared to the middle and lowest tertile, with a median survival of approximately 42.5 months and an AUC of 0.712. Accordingly, Mercier et al., 2018 reported that an increased platelet-neutrophil-to-lymphocyte ratio was linked to poor overall survival, with a median survival of 9.6 months in metastatic CRC patients (8). Yang et al. (2021) also conducted a study indicating that a high NLR correlated with poor progression-free survival, with a median of 6.1 months (21). These findings collectively suggest that neutrophil counts and related ratios serve as reliable predictors for CRC prognosis. Nonetheless, additional studies are necessary to comprehend their mechanistic role in CRC.

Tumor-associated neutrophils showed both pro- and anti-cancer effects, playing a dual role in both direct and indirect manners in the initiation and advancement of tumors (11). Tumors can stimulate increased production of neutrophils in the bone marrow and attract them to the tumor site (22). Once there, neutrophils are often polarized toward phenotypes that promote tumor growth and metastasis (23). Our linear and logistic regression analysis revealed a strong association between neutrophil levels and metastasis and tumor size, which aligns with previous research (24, 25). Studies have consistently highlighted the multifaceted role of neutrophils in various stages of the metastatic process, including creating a premetastatic niche and facilitating tumor cell invasion, migration, intravasation, and extravasation (24). Conversely, some findings emphasize a protective aspect of neutrophils against metastasis by activating and recruiting T cells and other leukocytes to the metastatic site (26).

Our research also emphasizes the importance of monocytes as a key predictor of mortality. In the case of CRC, monocyte counts have been identified as an independent prognostic factor for predicting the outcome. Higher absolute monocyte counts were significantly linked to poorer overall survival and progression-free survival outcomes (27). Furthermore, a meta-analysis revealed that distant metastatic status and increased absolute monocyte count were linked to worse outcomes in CRC patients (28). Cancer significantly impacts immune system functionality, reflected in altered immune cell counts, which serve as potential indicators of patient prognosis. Neutrophils play a key role in recruiting monocytes to the site of the injury. As a result of this process being disrupted, patients with tumors often have higher levels of immature neutrophils and monocytes in the bloodstream. These cells not only multiply in number but also move to the tumor’s environment, worsening local immune suppression (29).

We must acknowledge several limitations of our study. Its retrospective nature and single-center design may limit the generalizability of our findings. Nonetheless, our study offers valuable insights into the role of immune markers in CRC prognosis within a large cohort. Nevertheless, the variability in our results over time may be due to the lack of detailed data on treatment regimens, demographic characteristics, lifestyle factors, disease awareness, and screening practices—all of which are known to significantly influence survival outcomes. Additionally, a significant limitation of our study is the absence of mismatch repair (MMR) status data for a portion of our cohort, as routine testing for MMR was not consistently included in diagnostic protocols until 2014. This missing data may have influenced our ability to fully assess the relationship between MMR status and immune responses. Furthermore, we prioritized overall survival over progression-free survival (PFS) and recurrence data in our analysis due to the inconsistent availability of PFS and recurrence data across the patient cohort. Out of the patients in the study, only 133 (29.0%) were confirmed as positive for recurrence or progression, while 56 patients (12.2%) had negative outcomes, and 269 patients (58.7%) had indeterminate results. As overall survival was the most consistently documented outcome, it was the most suitable measure for our study. We acknowledge the limitation regarding the small number of stage IV patients in our study. This limited sample size does not allow us to perform a robust descriptive analysis of neutrophil counts as predictive values specifically for this subgroup. However, we acknowledge that this focus may limit our ability to thoroughly investigate the relationships between the biomarkers studied and cancer-specific outcomes such as PFS and recurrence. As a result, some nuances in the association between these biomarkers and disease progression may not be fully captured in our analysis. Therefore, we suggest that future studies explore the impact of these variables on CRC survival through prospective, larger, multicentric cohorts, as this will be crucial in validating and expanding upon our findings.

## Conclusion

In conclusion, our research suggests that higher neutrophil counts in patients with CRC can serve as potential risk factors for mortality and reliable prognostic indicators over a 5-year follow-up period. Our analysis identified neutrophils as the most significant predictors of overall survival. Additionally, our study brings attention to certain clinicopathological variables, such as metastasis and tumor size that can influence neutrophil counts, implying their role in these processes. These findings provide a foundation for creating personalized and effective prognostic tools in CRC management. Further research has the potential to improve our understanding of the prognostic role of neutrophils in CRC and could offer valuable insights into the mechanistic aspects of the development process of CRC.

## Data Availability

The raw data supporting the conclusions of this article will be made available by the authors, without undue reservation. Any private information will be removed to follow data privacy laws.

## Supplementary material

Supplementary Figure 1 Prognostic Value of Hematological Parameters and Clinicopathological Variables in Predicting Mortality in CRC. Cox regression analysis comparing Tertiles 1 (T1) and 2 (T2) versus Tertile 3 (T3) for **(A)** monocytes and **(C)** white blood cells. Receiver Operating Characteristic (ROC) curves demonstrate the predictive performance of **(B)** monocytes and **(D)** white blood cells, with corresponding Areas Under the Curve (AUC).
