# Corrigendum: Increased neutrophil counts are associated with poor overall survival in patients with colorectal cancer: a five-year retrospective analysis

**DOI:** 10.3389/fimmu.2025.1583828

**Published:** 2025-03-20

**Authors:** Libia Alejandra Garcia-Flores, María Teresa Dawid De Vera, Jesus Pilo, Alejandro Rego, Gema Gomez-Casado, Isabel Arranz-Salas, Isabel Hierro Martín, Julia Alcaide, Esperanza Torres, Almudena Ortega-Gomez, Hatim Boughanem, Manuel Macias-Gonzalez

**Affiliations:** ^1^ Department of Endocrinology and Nutrition, Virgen de la Victoria University Hospital, Málaga, Spain; ^2^ Institute of Biomedical Research in Malaga (IBIMA)-Bionand Platform, University of Malaga, Málaga, Spain; ^3^ Unidad de Gestión Clínica Intercentros (UGCI) de Anatomía Patológica, Instituto de Investigación Biomédica de Málaga (IBIMA), Hospital Universitario Virgen de la Victoria, Universidad de Málaga, Málaga, Spain; ^4^ Medical Oncology Service, Hospital Regional Universitario de Málaga, Biomedical Research Institute of Malaga (IBIMA), Málaga, Spain; ^5^ Unidad de Gestión Clínica Intercentros (UGCI) de Oncología Médica, Instituto de Investigación Biomédica de Málaga (IBIMA), Hospital Universitario Virgen de la Victoria, Málaga, Spain; ^6^ Centro de Investigación Biomédica en Red (CIBER) Fisiopatologia Obesidad y Nutricion (CIBEROBN), Instituto de Salud Carlos III, Madrid, Spain; ^7^ Lipids and Atherosclerosis Unit, Department of Internal Medicine, Hospital Universitario Reina Sofia, Cordoba, Spain; ^8^ Maimonides Institute for Biomedical Research in Cordoba (IMIBIC), Cordoba, Spain

**Keywords:** colorectal cancer, neutrophils, overall survival, prognosis, inflammation

In the published article, there was an error in the **Funding** statement. The phrase “a grant from ISCIII (PI18/01399, PI21/00633), UMA-FEDERJA-085” has been corrected. The correct **Funding** statement appears below.

