# Supplementary figures and images for: Increased neutrophil counts are associated with poor overall survival in patients with colorectal cancer: a five-year retrospective analysis
Source: Front Immunol. 2024 Sep 23;15:1415804. doi: 10.3389/fimmu.2024.1415804 (PMC11456424; doi:10.3389/fimmu.2024.1415804)

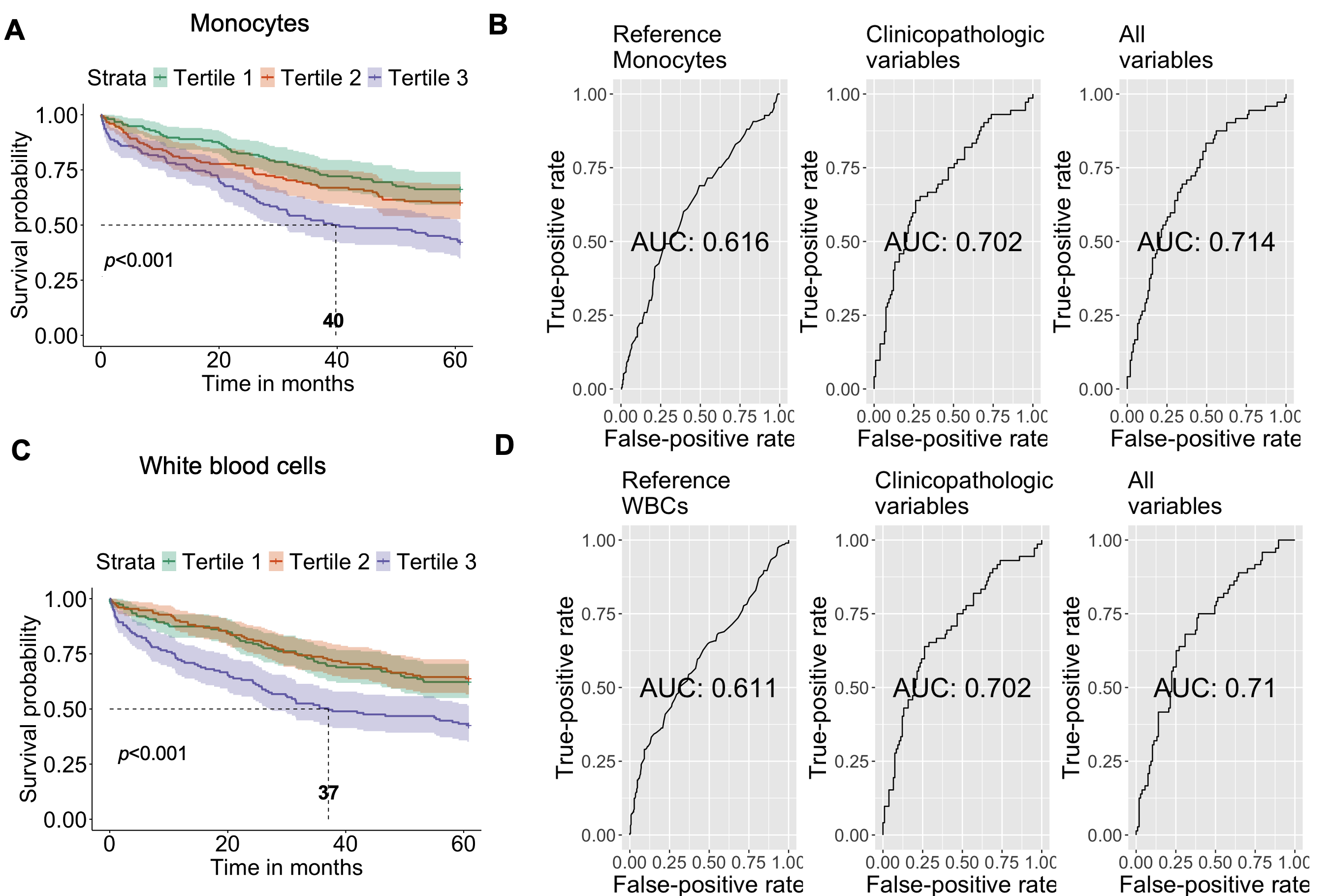

Supplement: Supplementary Figure 1 — Prognostic Value of Hematological Parameters and Clinicopathological Variables in Predicting Mortality in CRC. Cox regression analysis comparing Tertiles 1 (T1) and 2 (T2) versus Tertile 3 (T3) for (A) monocytes and (C) white blood cells. Receiver Operating Characteristic (ROC) curves demonstrate the predictive performance of (B) monocytes and (D) white blood cells, with corresponding Areas Under the Curve (AUC). [file Image1.jpeg]
