# Supplementary material for: Increased neutrophil counts are associated with poor overall survival in patients with colorectal cancer: a five-year retrospective analysis
Source: Front Immunol. 2024 Sep 23;15:1415804. doi: 10.3389/fimmu.2024.1415804 (PMC11456424; doi:10.3389/fimmu.2024.1415804)
Supplement: Supplementary file 2 [file Table1.docx]

**Supplementary Table 1.** Calculation of ratio formulas

| **NER** | Absolute Neutrophil Count 10³/µl / Absolute Eosinophil Count 10³/µl |
| --- | --- |
| **NLR** | Absolute Neutrophil Count 10³/µl / Absolute Lymphocyte Count 10³/µl |
| **NMR** | Absolute Neutrophil Count 10³/µl / Absolute Monocyte Count 10³/µl |
| **NPR** | Absolute Neutrophil Count 10³/µl / Absolute Platelet Count 10³/µl |
| **NWR** | Absolute Neutrophil Count 10³/µl / Total White Blood Cell Count 10³/µl |
| **NBR** | Absolute Neutrophil Count 10³/µl / Absolute Basophil Count 10³/µl |
| **PLR** | Absolute Platelet Count 10³/µl / Absolute Lymphocyte Count 10³/µl |
| **LMR** | Absolute Lymphocyte Count 10³/µl / Absolute Monocyte Count 10³/µl |
